# Structural basis for nucleotide-modulated p97 association with the ER membrane

**DOI:** 10.1038/celldisc.2017.45

**Published:** 2017-12-12

**Authors:** Wai Kwan Tang, Ting Zhang, Yihong Ye, Di Xia

**Affiliations:** 1 Laboratory of Cell Biology, Center for Cancer Research, National Cancer Institute, National Institutes of Health, Bethesda, MD, USA; 2 Laboratory of Molecular Biology, National Institute of Diabetes and Digestive and Kidney Disease, National Institutes of Health, Bethesda, MD, USA

**Keywords:** AAA protein, p97/VCP, IBMPFD/MSP, VIMP/SelS, p97-VIMP complex

## Abstract

Association of the cytosolic AAA (ATPases associated with various cellular activities) protein p97 to membranes is essential for various cellular processes including endoplasmic reticulum (ER)-associated degradation. The p97 consists of two ATPase domains and an N domain that interacts with numerous cofactors. The N domain of p97 is known to undergo a large nucleotide-dependent conformation switch, but its physiological relevance is unclear. Here we show p97 is recruited to canine ER membranes predominantly by interacting with VCP-interacting membrane protein (VIMP), an ER-resident protein. We found that the recruitment is modulated through a nucleotide-dependent conformation switch of the N domain in wild-type p97, but this modulation is absent in pathogenic mutants. We demonstrate the molecular mechanism of the modulation by a series of structures of p97, VIMP and their complexes and suggest a physiological role of the nucleotide-dependent N domain conformation switch. The lack of modulation in pathogenic mutants is caused by changes in interactions between the N and D1 domain, as demonstrated by multiple intermediate positions adopted by N domains of mutant p97. Our findings suggest the nucleotide-modulated membrane association may also have a role in other p97-dependent processes.

## Introduction

Protein quality control eliminates misfolded or unwanted proteins to maintain protein homeostasis and is an essential cellular process that has been identified in every subcellular compartment. In the endoplasmic reticulum (ER), an organelle that houses most nascent secretory and membrane proteins, misfolded or unwanted protein substrates are recognized by chaperones, directed to the ER membrane for retrotranslocation or dislocation to the cytosol, and subsequently targeted for degradation by the ubiquitin–proteasome system. This process has been dubbed ER-associated degradation (ERAD, for reviews, see Vembar and Brodsky [1] and Meyer and Weihl [2]). Retrotranslocation of substrates across the ER membrane requires the assembly of several large membrane protein complexes in a dynamic, yet poorly defined manner. In many cases, assembly of the mammalian ERAD apparatus is thought to be initiated by the association of luminal substrates with a member of the rhomboid pseudoprotease family consisting of Derlin-1, 2 and 3. This is followed by the recruitment of Hrd1, a ubiquitin ligase thought to form a retrotranslocation channel, and p97, an ATPase, together with its adaptor proteins Ufd1 and Npl4 (UN). Although the interaction of the p97-Ufd1-Npl4 complex with the ER membrane in mammalian cells appears to be mediated by several proteins residing in the ER membrane, including gp78, UbxD8, Erasin and Derlins [3], VCP-interacting membrane protein (VIMP) was identified as a major adaptor for p97 in studies using canine pancreas ER membranes [4–6].

Mammalian p97 (also called valosin-containing protein or VCP, cdc48 in yeast, and Ter94 in *Drosophila*) is a highly conserved AAA (ATPases associated with various cellular activities) protein with a homo-hexameric ring structure; each p97 subunit is composed of two AAA ATPase domains (D1 and D2) preceded by an N-terminal domain (N domain) [7]. ATP hydrolysis of p97 is required for the translocation of ERAD substrates from the ER to the cytosol [8]. The essential role of p97 in ERAD has been further recognized by mutations identified from patients with multisystem proteinopathy 1 (MSP1), also known as inclusion body myopathy associated with Paget disease of the bone and frontotemporal dementia (IBMPFD), which causes accumulation of ERAD substrates [9, 10]. Although the ATPase activity of the D2 domain has been associated with substrate unfolding activity [11, 12], the function of the ATPase activity of the D1 domain remains unclear, even though the D1 domain was shown to control the conformation switch of the N domain [13], which may also involve the release of polyubiquitinated substrates [11]. Curiously, despite the lack of any transmembrane domains, a significant portion of p97 is found to be associated with cellular membranes [14–17]. This association allows p97 to gain access to membrane substrates as in ERAD and other membrane-associated degradation processes, but how this process is regulated is unclear.

The subcellular localization of p97 appears to be regulated by a large number of p97-interacting adaptor and/or cofactor proteins, through which it exerts diverse functions in different cellular pathways [8, 18–22]. More than 30 cofactors and adaptors have been identified; most of these proteins interact with the N domain of p97 via several conserved binding motifs [23–28]. Interestingly, although for some well-studied adaptor proteins such as p47, Ufd1-Npl4 and FAF1, a 1:1 binding stoichiometry was observed between these adaptor proteins and isolated p97 N domains, a lower stoichiometry of 1−3 molecules of adaptor proteins per hexameric p97 is often seen [25, 29, 30], suggesting the presence of a mechanism in full-length p97 to limit the number of bound adaptor proteins.

VIMP, also known as selenoprotein S, is a 21 kDa ER membrane protein. It is predicted to span the ER membrane once with a short luminal segment, and has a long C-terminal cytosolic segment of 140 residues [31]. The cytosolic segment of VIMP consists of an N-terminal half (residues 51−122) of two helices (PDB:2Q2F, unpublished) and an intrinsically disordered C-terminal half [32]. Toward the C-terminal end of the polypeptide is the selenocysteine residue (SeC188), which gives VIMP its seleno-dependent oxidoreductase activity and presumably provides protection for cellular oxidative stress [32, 33]. VIMP, together with Derlins, is thought to contribute to the association of the p97-Ufd1-Npl4 complex with the ER membrane, forming an ER membrane-associated module in ERAD [4–6, 34]. A putative 11-residue (R*X*_*5*_AA*X*_*2*_R) VCP-interacting motif (VIM) was identified at the helical region (residues 78–88) of VIMP [35] and is similar to the VIM peptide in the ubiquitin ligase gp78, which is known to bind to the N domain of p97 [36]. However, inconsistencies exist in the literature with regard to the exact location of the VIMs for VIMP. In one case, the region consisting of residues 50−71 was proposed for p97 binding based on an nuclear magnetic resonance (NMR) study [32]. In another, the C-terminal disordered region of VIMP was shown to be critical for p97 interaction [37]. Thus, the controversy over the precise sequence motif that binds p97 in VIMP needs to be resolved. Furthermore, whether or not p97 membrane recruitment is linked to its ATPase cycle and how disease-associated p97 mutations affect this regulation remain unclear.

Here we show that VIMP has an essential role in recruiting p97 to the ER membrane. The recruitment of p97 is modulated by the nucleotide state of p97. In p97 mutants, however, this modulation is diminished. We further provide evidence by determining the structures of p97 in complex with VIMP to explore the molecular basis of this nucleotide-dependent modulation. Our results reveal that an N domain conformation switch in p97 might drive a cycle of p97 membrane attachment and detachment, which does not occur in pathogenic mutants. The defective function of mutant p97 is likely caused by altered interactions between the N and D1 domains, as evidenced by multiple intermediate positions of the N domains in the structures of the p97-VIMP complex, which were captured when mutants were studied.

## Results

### VIMP is the p97 receptor on canine ER membranes

**Figure 1:**
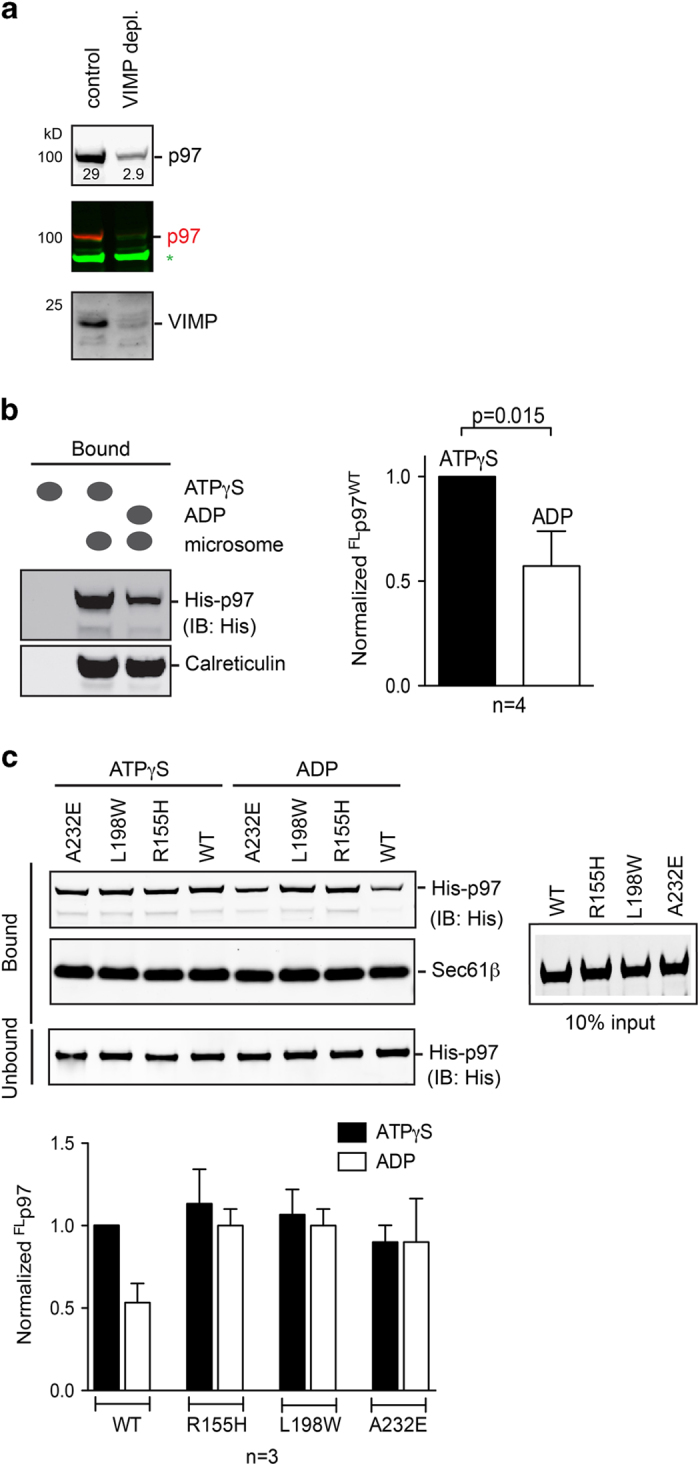
VIMP-mediated ER membrane association of p97. (**a**) Co-depletion of p97 with VIMP. DeoxyBigCHAP (1%) solubilized canine pancreas microsomes were centrifuged. The supernatant was incubated with Protein A beads containing anti-VIMP antibodies three times. Immunoblotting of the unbound fractions showed the co-depletion of ER membrane-associated p97. The top panel shows the amount of p97 before VIMP depletion. The middle and bottom panels show the amount of remaining p97 and VIMP after VIMP depletion, respectively. A nonspecific protein band labeled * was used as a loading control. (**b**) Differential binding of ^FL^p97^wt^ to ER microsomes probed by anti-His tag antibody in the presence of 2 mM ATPγS or ADP. Calreticulin, a protein specific to the ER, was used as an ER marker. (**c**) Binding of three ^FL^p97^mt^ to ER microsomes in the presence of ATPγS or ADP.

As part of an ER membrane protein complex that mediates retrotranslocation, VIMP was shown previously to interact with p97 in conjunction with Derlin-1 and this interaction was found to be independent of Ufd1-Npl4 [34]. VIMP was further suggested to bridge the interaction between Derlin-1 and p97 on the basis of intracellular expression of these components [34]. To eliminate the possibility that these observed interactions might be the result of intracellular protein overexpression, we carried out an *in vitro* VIMP depletion experiment. Using canine pancreas microsomes, we found that the amount of p97 associated with microsomes was dramatically reduced (by as much as 90%) as a result of co-depletion when VIMP was depleted biochemically (Figure 1a). This was in comparison with control microsomes, in which both VIMP and p97 protein were detected (Figure 1a). This result is consistent with a previous finding that the complex of Derlin-1 and VIMP was the major component co-precipitated with p97 from these membranes, further supporting VIMP as a major mediator of p97 membrane association in the context of pancreatic ER membranes.

### Association of wild-type full-length p97 with the ER membrane is modulated by ATP

As the N domain of p97 undergoes nucleotide-dependent conformational change [13, 38–40], we asked whether the membrane recruitment of p97 is sensitive to the types of nucleotides present. Employing a membrane-binding assay, we tested this idea by incubating isolated ER microsomes with purified full-length wild-type p97 (^FL^p97^wt^) in the presence of either ADP or the non-hydrolysable ATP analog ATPγS. The microsomes were subsequently sedimented by ultracentrifugation. Immunoblotting analyses showed consistently that a significantly lower amount of ^FL^p97^wt^ bound to the membranes in the presence of ADP than in the presence of ATPγS (Figure 1b), suggesting nucleotide-dependent modulation of membrane binding. In the presence of ATPγS, pathogenic p97 variants (^FL^p97^mt^) bearing MSP1-associated mutations (R155H, L198W or A232E) bound to microsomes with similar efficiency to ^FL^p97^wt^ (Figure 1c), which was largely unaffected by the presence of ADP (Figure 1c). These results suggest that the association of ^FL^p97^wt^ to the ER membrane is modulated by ATP, whereas in pathogenic mutant p97, this modulation is abolished.

### Binding of ^FL^p97^wt^ to VIMP is also modulated by ATP

**Figure 2:**
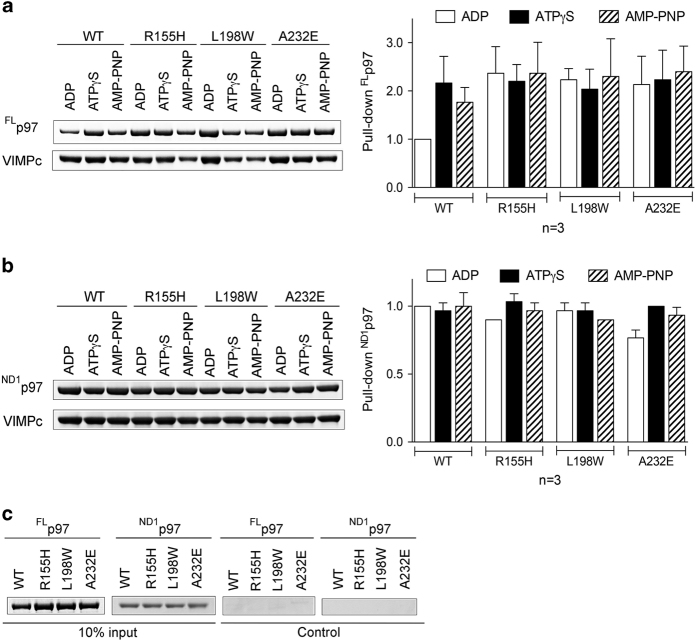
Interaction of p97 variants and VIMPc. GST-tagged VIMPc was used to pull-down p97 and its variants. Purified ^FL^p97 or ^ND1^p97 (wild-type or pathogenic mutants R155H, L198W or A232E) was incubated with VIMPc in the presence of different nucleotides, as labeled, followed by washing. The glutathione-conjugated beads were subsequently analyzed by sodium dodecyl sulfate–polyacrylamide gel electrophoresis (SDS-PAGE). (**a**) A representative result of the pull-down assays showing the nucleotide-sensitive interaction of VIMPc with ^FL^p97^wt^, but not with pathogenic ^FL^p97^mt^. Three repeats of the pull-down assays were done for each p97 variant and results are plotted as a bar representation normalized to ^FL^p97^wt^ in the presence of ADP. (**b**) A typical result of the pull-down assays with ^ND1^p97 variants showing they interact with VIMPc under all conditions. Three repeats of the pull-down assays were done for each ^ND1^p97 variant and results are plotted as bar representations normalized to the ^ND1^p97^wt^ in the presence of ADP. (**c**) Controls for the pull-down experiment showing 10% of the input p97 protein (left two panels); and control experiments in the absence of the GST-tagged protein (right two panels).

As VIMP is a major interaction partner for p97 in canine pancreas microsomes, we tested whether the interaction of p97 with VIMP could be modulated by ATP using isolated protein components. As the cytosolic segment of VIMP (VIMPc, residues 49−187) was previously shown to be responsible for interacting with the N domain of p97 [34], we first used Glutathione S-transferase (GST)-tagged VIMPc to pull-down ^FL^p97^wt^ or ^FL^p97^mt^ in the presence of ADP, ATPγS or AMP-PNP (Figure 2a and c). Consistent with the results of the membrane-binding assay, ^FL^p97^wt^ maintained a strong interaction with VIMPc in the presence of ATPγS or AMP-PNP but this interaction weakened significantly in the presence of ADP (Figure 2a and c). By contrast, this nucleotide-regulated interaction was not observed for various ^FL^p97^mt^, as they showed similar binding efficiency to VIMPc, regardless of the nucleotide present (Figure 2a and c).

### A minimal fragment containing 40 residues of VIMP is required for interaction

**Figure 3:**
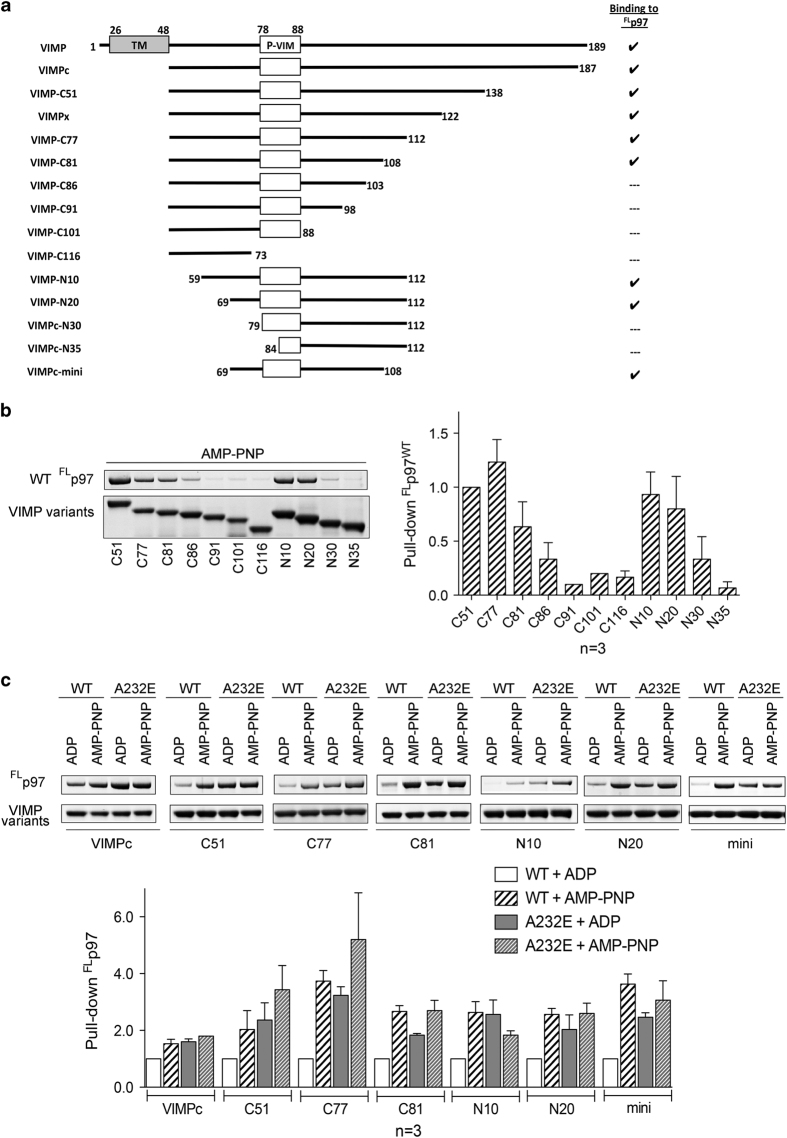
Identification of the minimal p97-interacting fragment for VIMP. (**a**) Schematic diagram showing different constructs of VIMP used in the present study. The predicted transmembrane (TM) segment and the putative VCP-interacting motif (P-VIM) are represented in boxes. Those that bind to p97 are check-marked in the column on the right. (**b**) Representative result of the pull-down assays performed in the presence of 2 mM AMP-PNP showing the interactions or lack of interaction between ^FL^p97^wt^ and GST-tagged VIMP fragments. Three repeats of the pull-down assays were performed for each VIMP fragment and results are plotted as bar representations normalized to the VIMP fragment C51. (**c**) The pull-down assay of VIMP variants with ^FL^p97 (wild-type and pathogenic mutant A232E) in the presence of indicated nucleotides.

To resolve the inconsistencies in the literature with respect to the region of VIMP interacting with p97 [32, 37] and the actual p97-interacting sequence, we determined the minimal VIMP segment required for the interaction. A series of GST-VIMPc fragments were generated (Figure 3a) with deletions from either the C- or N-terminus and were used to pull-down ^FL^p97^wt^ (Figure 3b). We found that the smallest fragment of VIMP capable of interacting with p97 consists of 40 residues (A69-E108). Compared with the putative VIM (P-VIM) sequence (R*X*_*5*_AA*X*_*2*_R), this minimal binding motif contains 20 additional residues C-terminus to the P-VIM. The nucleotide-sensitive interaction between ^FL^p97^wt^ and VIMP is preserved among all VIMP fragments that are capable of binding ^FL^p97^wt^ (Figure 3c).

### Structure of VIMP consists of two helices jointed with a flexible elbow

**Figure 4:**
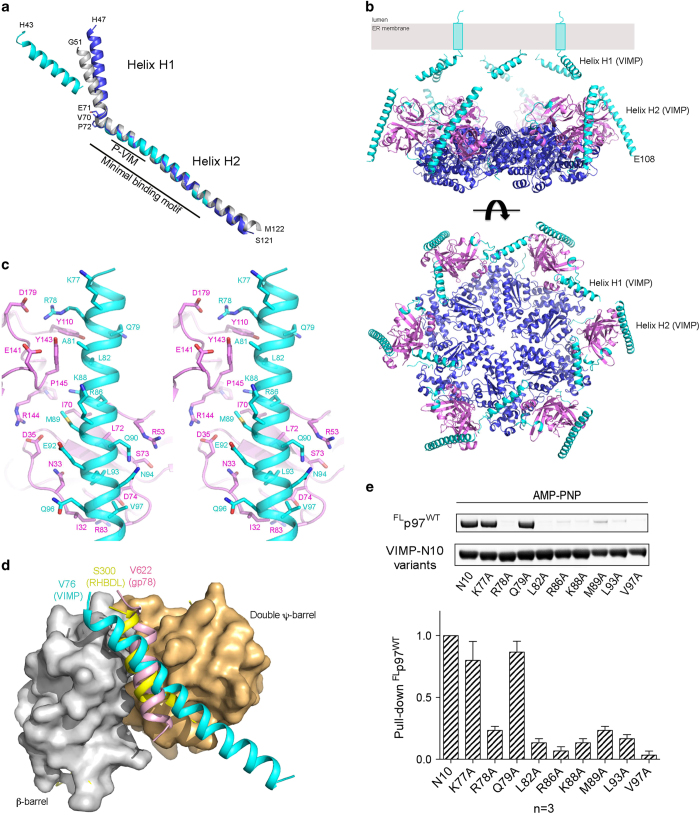
Crystal structures of VIMPx and its complex with p97. (**a**) Superposition of the structures of VIMPx alone (blue) and chain D of ^ND1^p97^L198W^-VIMPx (cyan) with PDB: 2Q2F (gray) based on residues 74−115 in helix H2. The putative VCP-binding motif (P-VIM, residues 78−88) and the minimal binding motif (residues 69−108) identified in the present study are labeled. The elbow angles between helixes H1 and H2 were determined to be 132.9^o^, 142.2^o^ and 142.7^o^ for VIMPx alone, PDB:2Q2F, and chain D of ^ND1^p97^L198W^-VIMPx, respectively. The chain D of the ^ND1^p97^L198W^-VIMPx complex appears to have a helix unwinding near the elbow. (**b**) Cartoon representation of the hexameric structure of the ^ND1^p97^L198W^-VIMPx complex. With AMP-PNP bound at the D1 domain, the N domain of ^ND1^p97^L198W^ is in the Up-conformation. The side and top views of the hexameric complex are shown in the upper and lower panels, respectively. The N domain is colored magenta, D1 domain blue and VIMPx cyan. (**c**) Stereoscopic pair showing details of the VIMPx binding environment. VIMPx binds to the surface located between the two subdomains of the N domain of p97. The N domain and VIMPx are represented by magenta and cyan ribbons, respectively. Residues contributing to the binding interface are shown in stick models and labeled. (**d**) Comparison of the interactions of p97 N domain with helical VCP-interacting motifs (VIMs) from various adaptor proteins. The N domains were superposed and are shown as a surface representation in light brown and gray for the double ψ-barrel and β-barrel subdomains, respectively. The VIM peptide of gp78 (PDB:3TIW) [36] and VBM (VCP-binding motif) peptide of rhomboid protease RHBDL4 peptide (PDB:5EPP) [28] are shown as pink and yellow short helices fitting into the groove between the two subdomains of the N domain, whereas the VIMPx in the structure of ^ND1^p97^L198W^-VIMPx (cyan) is depicted as a long helix deviating significantly in its binding orientation from those of the other binding partners. (**e**) Pull-down assay showing the effect of mutations to the binding interaction between ^FL^p97^wt^ and GST-tagged VIMP-N10 variants. The assay was performed in the presence of 2 mM AMP-PNP.

The C-terminal portion (residues 123−189) of VIMP is highly disordered with no apparent secondary structures, as shown by an nuclear magnetic resonance (NMR) study [32]. We also showed that the segment of VIMP in the residue range 69−108 is essential for interacting with p97. We therefore used the fragment of VIMP encompassing the minimal p97-interacting motif (residues 49−122, VIMPx) to investigate the structural basis of its interaction with p97. First, we obtained crystals of VIMPx alone, which diffracted X-rays at synchrotron to about 2 Å resolution. The structure was solved by molecular replacement using a deposited structure (PDB:2Q2F, residues 51−122, unpublished) as a search model and refined to 2.2 Å resolution (Table 1). The structure of VIMPx consists of two long α-helices (H1 and H2) with an elbow bend at residues V70-P72 forming an elbow angle of 132.9º (Figure 4a). The minimal p97-interacting fragment (residues 69−108) we identified is located in helix H2, immediately following the bend between the two helices, overlapping with the P-VIM (residues 78−88) [36] (Figure 4a).

Superposition of the VIMPx structure with PDB:2Q2F revealed a large root-mean-square deviation of 1.25 Å using 65 superposed Cα atoms. The main difference between the two structures is the elbow angle, which is 10° larger for PDB:2Q2F, suggesting that a degree of flexibility exists between the two α-helices H1 and H2.

### Structures of the hetero complex between pathogenic p97 and VIMPx

It is well established that isolated hexameric ^FL^p97^wt^ has prebound or occluded ADP closely associated with the D1 domains of a subset of subunits, leading to asymmetry in subunit conformations and difficulty in crystallization in the presence of ATP analogs [13, 41, 42]. Taking advantage of the pathogenic p97 mutants that are able to achieve uniform subunit conformation in the presence of ATP analogs, we successfully obtained crystals of VIMPx in complex with two ^ND1^p97 mutants (residues 1−460), each carrying a single pathogenic mutation either in the N-D1 linker region (L198W, ^ND1^p97^L198W^) or in the D1 domain (A232E, ^ND1^p97^A232E^), in the presence of the non-hydrolyzable ATP analog AMP-PNP. Structures were solved and refined to resolutions of 3.41 Å and 2.79 Å for the ^ND1^p97^A232E^ and ^ND1^p97^L198W^ complexes, respectively (Table 1).

The structural solution of ^ND1^p97^A232E^ alone allowed calculation of a difference Fourier map that revealed additional electron densities attributable to both AMP-PNP and VIMPx (Supplementary Figures S1a and b). For the first time, the AMP-PNP moiety bound at the D1 nucleotide-binding pocket was observed in a p97 crystal structure. The binding of AMP-PNP causes the N domain to move above the D1 ring (Figure 4b), which was analogous but not identical to the Up-conformation previously observed in the presence of ATPγS [13] (see Discussion section). To obtain an anchor point for sequence assignment of VIMPx in the complex, we also expressed and purified selenomethionine (SeMet)-derivatized VIMPx (^SeMet^VIMPx) for co-crystallization with ^ND1^p97^A232E^ (Table 1). The anomalous difference Fourier map identified a large peak in the middle of the tubular VIMPx electron density (Supplementary Figure S1c), which was assigned to M89. Based on the position of M89, we built 33 residues (76−108) of VIMPx into the density, corresponding to helix H2 of VIMPx (Supplementary Figure S1d). This assignment agrees well with the result from the determination of the minimal fragment required for p97 interaction. The refined structure achieved an R_*work*_ and R_*free*_ of 19.0 and 25.3, respectively.

A second complex between the p97 mutant L198W (^ND1^p97^L198W^) and VIMPx was crystallized. This crystal has a larger unit cell (Table 1) sufficient for two ^ND1^p97^L198W^ subunits (chains A and B) and two VIMPx molecules (chains C and D) in a crystallographic asymmetric unit, which, after symmetry expansion, represent two separate hexameric rings packed back-to-back in the crystal lattice (Supplementary Figure S1e). AMP-PNP moieties were found bound to the D1 domains of both subunits. A difference Fourier map showed densities corresponding to VIMPx molecules binding to each p97 subunit. A 38-residue fragment (residues 73−110) representing helix H2 was assigned to the VIMPx bound to chain A, and the density associated with chain B was fit with a 61-residue model (residues 43−65, 71−109) that included both helices H1 and H2 (Figure 4b). Inclusion of VIMP models led to successful crystallographic refinement of the structure, giving rise to an R_*work*_ and R_*free*_ of 23.3 and 29.3, respectively (Table 1). It is worth mentioning that the two crystal forms obtained here resulted from differing amounts of ethanol present in the crystallization conditions rather than a difference in the mutation site, as the L198W mutant can be crystallized in both forms.

The H1 helix in the longer VIMP model of the ^ND1^p97^L198W^-VIMPx crystal is stabilized by interactions with an N domain of a neighboring molecule in the crystal (Figure 4b and Supplementary Figure S1f). Although the elbow angle (142.7°) between helices H1 and H2 in the complex (D chain in the ^ND1^p97^L198W^-VIMPx) is not very different from those (132.9° and 142.2°) found in the VIMP-alone structures (Figure 4a), the directions of the H1 helix (defined by the swing angle) in these structures are different, further supporting the notion that there is an intrinsic flexibility built into the H1-H2 joint.

### Structural basis of p97 binding to VIMP

Figure 4c is derived from the highest resolution structure of the p97-VIMP complex (^ND1^p97^A232E^-VIMPx), revealing detailed interactions of the two molecules with the following features: (1) binding to p97 is exclusively mediated via helix H2 of VIMP, which is consistent with the experimentally determined minimal fragment of VIMP required for binding. (2) On the p97 side, the VIMP-binding surface covers a much larger area of the N domain, when compared with previously reported ^N^p97-peptide complexes (isolated N domain of p97 in complex with the VIM of ubiquitin ligase gp78 [36] or with the VCP-binding motif (VBM) of rhomboid protease RHBDL4 [28]. Although in all three structures, a single helix is found to interact with the N domain, each binds to a different surface on the N domain, interacting with a different set of residues (Figure 4d). Although the gp78 and RHBDL4 peptides efficiently transverse the surface between the two subdomains of the p97 N domain [36], the binding of the VIMP helix is tilted, spanning a much larger area of the subdomain interface, resulting in interactions with a different set of residues. (3) Binding between the two molecules is mediated by charged, H-bonding and hydrophobic interactions with a buried surface area of 759 Å^2^. (4) The orientation of VIMPx bound to p97 agrees with its topological position in the ER membrane, as the transmembrane helix immediately before the N-terminus of VIMPx would be in the open space above the hexameric p97 ring, where the lipid bilayer of the ER membrane would be located (Figure 4b).

To ascertain the actual binding surface of VIMP, we substituted alanine for individual residues that interface with p97 and determined the effect on binding in the presence of AMP-PNP using the GST pull-down assay (Figure 4e). Mutations of residues (R78A, L82A, R86A, M89A, L93A and V97A in Figure 4c and Supplementary Figure S1d) that directly face p97, including the two residues within the P-VIM (R87A and K88A), showed significantly weakened binding with ^FL^p97. Mutation of the large residue K88 that is to one side of the binding interface also showed reduced binding, suggesting contributions of this residue to the binding of p97. By contrast, residues such as K77 and Q79 that have no interactions with p97 in the complex structure displayed no effect on binding in our assay when mutated to alanine. Thus, the structure of the complex maps out a strip on the surface of the N domain for interaction with VIMP (Figure 4d and Supplementary Figure S1c) and most of the contact surface is provided by the N-terminal double β-barrel subdomain with small contribution from the C-terminal β-barrel subdomain, making contacts with the N-terminal part of VIMP.

**Figure 5:**
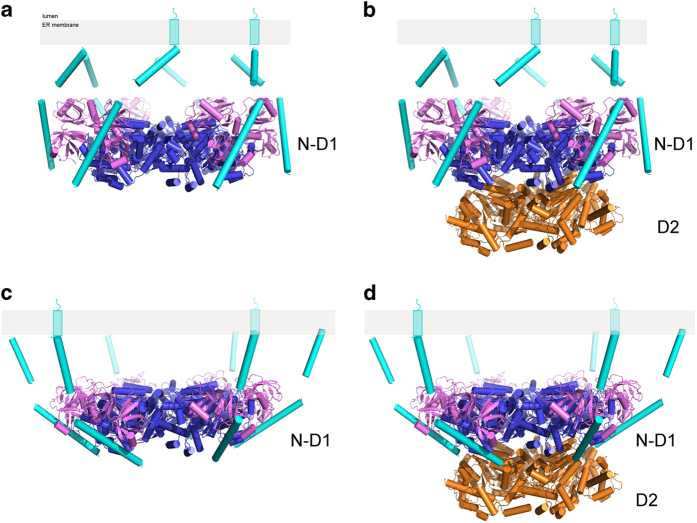
Molecular modeling suggests a mechanism for ATP-dependent VIMP binding to subunits of p97. The structure of VIMP is represented by cylinders colored cyan. The structure of p97 is shown as a cartoon diagram with the N domain in magenta, the D1 domain in blue and the D2 domain in coral. (**a**) Experimental structure of VIMP in complex with the N-D1 domain of p97 in the presence of AMP-PNP, in which the N domains are in the Up-conformation. (**b**) Modeling of VIMP bound to full-length p97 in the presence of AMP-PNP, in which the N domains are in the Up-conformation. (**c**) Modeling of VIMP bound to the N-D1 fragment of p97 in the presence of ADP, in which the N domains are in the Down-conformation. (**d**) Modeling of VIMP bound to full-length p97 in the presence of ADP, in which the N domains are in the Down-conformation.

Using the present structures of the ^ND1^p97^L198W^-VIMP complex bound with AMP-PNP and of ^FL^p97^wt^ (PDB:3CF3) [43] bound with ADP, we modeled ^FL^p97 and ^ND1^p97 in two conformations. In one conformation with AMP-PNP in the D1 domain, the N domain adopts an Up-conformation with its VIMP-binding surface facing outward (Figure 5a and b), allowing VIMP to bind to the periphery of the p97 hexameric ring with the transmembrane region positioned above the N-D1 ring. In this configuration, the bound VIMP does not cause steric clashes with the p97 hexamer, in either ^FL^p97 or ^ND1^p97. By contrast, in the second conformation with bound ADP in the D1 domain, the N domain is in the Down-conformation, resulting in the VIMP-binding surface facing downward and placing helix H2 of VIMPx below the D1 ring. Although no steric clashes were found between VIMP and ^ND1^p97 (Figure 5c), the helix H2 of VIMPx, tucked under the D1 ring, has serious steric clashes with the D2-ring of ^FL^p97 (Figure 5d). This model predicts that the nucleotide-dependent regulation of VIMPx–p97 interaction should be dependent on the presence of the D2 domain. Indeed, when N-D1 fragments of p97 (^ND1^p97^wt^ or ^ND1^p97^mt^) were used in the pull-down assay, all ^ND1^p97 variants bound to VIMPc similarly, regardless of the type of nucleotide present (Figure 2b and c). This conclusion is further supported by isothermal titration calorimetry experiments, showing similar K_d_ values for binding full-length, N-D1 fragment, or N domain alone of wild-type p97 with VIMPx (Supplementary Table S2).

## Discussion

### Multiple intermediate positions adopted by N domains suggest a weakened interaction between N and D1 domains in p97 pathogenic mutants

**Figure 6:**
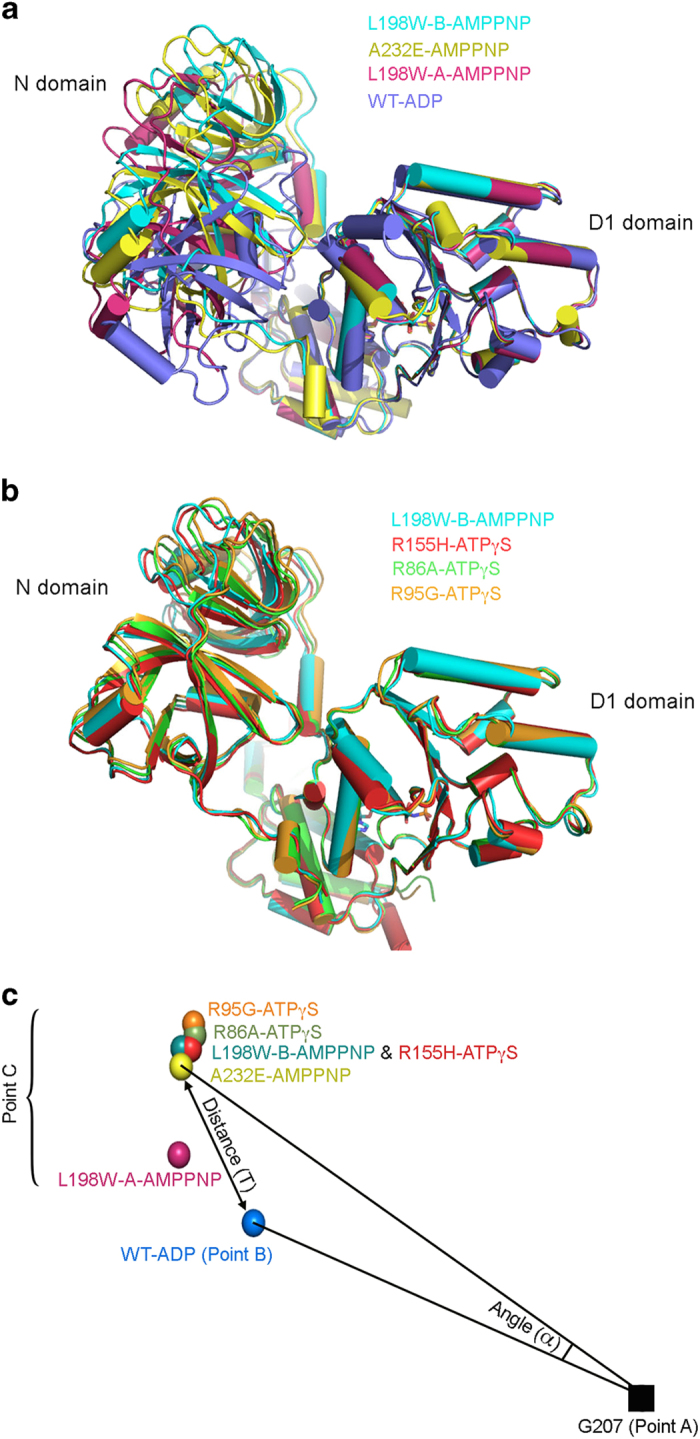
Trajectory of N domain movement revealed by ^ND1^p97 structures bound with different nucleotides. (**a**) Superposition of ^ND1^p97 structures bound with AMP-PNP and ADP. The three independently determined AMP-PNP-bound p97 structures solved in the present study were superposed with the ADP-bound structure (PDB:1E32 [55]) based on the D1 domain. Chain A (magenta) and chain B (cyan) of ^ND1^p97^L198W^-VIMPx, ^ND1^p97^A232E^-VIMPx (yellow) and PDB:1E32 (blue) are represented in cartoon diagrams. (**b**) Superposition of ^ND1^p97 structures with bound ATPγS and AMP-PNP. Based on the D1 domain, chain B of ^ND1^p97^L198W^-VIMPx (cyan) was superposed with three ATPγS-bound structures: ^ND1^p97^R155H^ (PDB:4KO8 [44]) in red, ^ND1^p97^R86A^ (PDB:3HU2 [13]) in green and ^ND1^p97^R95G^ (PDB:3HU1 [13]) in gold, and shown as cartoon diagrams. (**c**) A schematic diagram showing the center of mass (COM) positions of the N domains corresponding to different nucleotide states of the D1 domain. All structures were superposed based on the D1 domain (residues 210−370), and the COMs calculated for the N domains (residues 30−180) in different states are shown as spheres in different colors. Points A and B of the triangle represent two anchor points: A for the Cα atom of G207 (black square) and B for the COM of the N domain in the Down-conformation represented by PDB:1E32. Points C are COMs of N domains corresponding to different nucleotide states of the D1 domain. The distance of movement (T) is a measure of the length from Point B to various Points C; and the angle (α) of the N domain movement has a unit in degrees.

Why do p97 pathological mutants lose nucleotide-dependent regulation of VIMP binding? Visual inspection suggested that the two subunits of the ^ND1^p97^L198W^-VIMPx complex in the asymmetric unit have different N domain conformations. Indeed, pair-wise superposition of the D1 domains from all three AMP-PNP-bound N-D1 structures leads to significant positional and rotational deviations of the N domains (Supplementary Table S1 and Figure 6a and b). To quantify the movement of the N domain in response to different nucleotide states of the D1 domain, we constructed a triangle linking a fixed point A (G207) in the D1 domain to the center of mass of the N domain in the Down-conformation (point B, PDB:1E32) and to the center of masses of the N domains in various Up-conformations (point C, Figure 6c), which allowed us to compute the swing angle (α) between lines AC and AB and the distance of movement (T) of the N domain in reference to the Down-conformation defined by PDB:1E32 (Table 2). Among the three nucleotide states, the movement of the N domain was the greatest in the presence of ATPγS, with a mean α=21.5^o^, T=12.2 Å from the Down-conformation, whereas in the presence of AMP-PNP, the N domain adopted various intermediate conformations, with a wide range of distances of movement T (6.4 Å, 11.3 Å and 9.6 Å).

As AMP-PNP and ATPγS are both ATP analogs, we expected to see the N domains adopt the Up-conformation in the presence of AMP-PNP, as shown in the ATPγS-bound structure. The fact that the N domains in these AMP-PNP-bound structures of ^ND1^p97^mt^-VIMP complexes take three different positions or conformations was a surprise (Figure 6a and c). Given the pull-down results showing that the interactions between VIMP and ^FL^p97^wt^ are significantly reduced in the presence of ADP compared with AMP-PNP (Figure 2a and c) and those between VIMP and ^FL^p97^mt^ under the same conditions remain unchanged, we concluded that the interactions between the D1 and N domains are weakened in mutant p97. This conclusion is consistent with the observation that all MSP1 or IBMPFD mutations are located at the interface between the N and D1 domains.

### Role of D1 domains in modulation of p97 association with the membrane

In this work, we have presented the structures of ^ND1^p97^mt^-VIMPx complexes in the presence of AMP-PNP, in which the N-D1 fragment used is the so-called short form containing 460 residues (1−460). This is in contrast to the longer form containing 480 residues (1−480) that we previously used [13, 42, 44]. Although there are reported differences between the short and long forms of N-D1 fragments in terms of ATPase activities and asymmetry in subunit organization [42, 45], both forms can form complexes with VIMPx. Crystals of VIMPx in complex with the longer version of the N-D1 fragment were also obtained and diffracted X-rays to better than 2.8 Å resolution (Supplementary Table S3). Thus, there is no apparent difference between the short and long forms of the N-D1 fragment with respect to its nucleotide sensitivity in VIMP binding.

We noticed that membrane association or VIMP binding was not completely abolished by ^FL^p97^wt^ in the presence of ADP, using either ER microsomes or isolated VIMPx, (Figures 1b, c and 2a). In either case, a significant portion of ^FL^p97^wt^ remains attached to the membrane or bound to VIMPx. Thus, the hexameric ^FL^p97^wt^ is capable of interacting with VIMPx even under the ADP conditions, albeit at a low level.

In an earlier study, we proposed that the D1 domain of an ^FL^p97^wt^ subunit has four nucleotide states: an ATP state, ADP-locked state, ADP-open state and empty state [13]. The presence of an ADP-locked state is supported by the prebound or occluded ADP that was co-purified with the protein, difficult to remove, and exclusively associated with the D1 domains [13, 41, 44]. We also proposed that the ADP-locked state has to be converted to an ADP-open state before nucleotide exchange. The hallmark of ADP-open state is its weakened interactions between the N and D1 domains. The presence of the ADP-open state is supported by the observations that in MSP1 or IBMPFD mutants the prebound ADP can be replaced by ATP [44].

For both isolated ^FL^p97^wt^ and ^ND1^p97^wt^, the amount of co-purified or occluded ADP in the D1 domains is approximately 3−4 molecules per hexamer [41, 44]. These subunits are in the ADP-locked state and not available for VIMP binding by ^FL^p97^wt^. In the presence of ADP, the empty sites are occupied by ADP and are in the ADP-open state, which permits VIMP binding and is likely the reason for the observation of VIMP binding in the presence of ADP (Figures 1 and 2). This strongly supports our hypothesis of the existence of a balance or equilibrium between the ADP-locked and ADP-open states for wild-type p97 [13]. This balance is tipped in the IBMPFD mutants in favor of the ADP-open state, which explains the loss of nucleotide-sensitive binding to VIMP or microsomal membranes for the three mutants. Mechanistically, the tightly controlled ADP-locked state allows for more precise control over the movement of the N domain.

### Implications concerning ERAD and defects in ERAD because of MSP1 mutations

Our present work demonstrates, both with isolated proteins and with canine pancreas ER microsomes, that VIMP is a major adaptor that recruits p97 to the ER membrane under certain conditions (Figure 1a), although we cannot exclude other ER membrane receptors such as gp78, Hrd1, Derlins and UbxD8 having a similar or redundant role. The regulation of p97 recruitment to the ER membrane is likely affected by the relative abundance, affinity, and conformational dynamics of these adaptors in a tissue-specific manner. In fact, reducing the level of VIMP by CRISPR in HEK293 cells does not significantly affect p97 association with the ER membrane (data not shown). Nevertheless, our experiments suggest that in addition to the expression level of adaptors, the amount of ER-associated p97 could also be modulated by the ATP hydrolysis cycle of p97. The role of VIMP in recruiting p97 may have evolved for two reasons: it allows a timely and specific response to ER stress, as VIMP is a constituent of the ER. Second, the nucleotide-modulated attachment and detachment of p97 from VIMP and thus the ER permits a reversible mechanism to engage p97 function. This model may be applicable to other p97-dependent processes such as mitochondrion-associated degradation [46]. Clearly, performing these site-specific functions requires site-specific localization of p97, which can be achieved via site-specific adaptors. Consistent with this view, a comparison of the structures of ^ND1^p97-VIMPx complex with ^N^p97-VBM or ^N^p97-VIM illustrates convincingly that similar helical VIMs in VIMP, RHBDL4 and gp78 can interact with different surfaces of the N domain.

Our study raises the question of whether the observed nucleotide-dependent interaction of p97 with VIMP can serve as a general paradigm for p97–adaptor interaction. Support for this model has been found with respect to other p97 adaptor proteins such as p47 and p37, which not only show nucleotide-dependent binding affinity changes but also display altered ATPase activity of p97 upon interaction [47–49]. Recently, it was also shown that the binding of the ERAD-specific cofactors Ufd1/Npl4 (UN) is able to influence the intrinsic ATPase activity of p97 [11, 12]. The binding of VIMP, on the other hand, does not appear to have an influence on the ability to p97 to hydrolyze ATP (Supplementary Figure S2). We also tested the nucleotide dependency of p97 binding with SVIP (via VIM motif), but did not observe any discernable difference in binding in the presence of different nucleotides using a pull-down assay. Thus, the phenomenon of nucleotide-dependent interaction only applies to a certain subset of p97 adaptors/cofactors.

Our new data are also consistent with the role of the D1 domain in regulating p97 function. Such regulation includes but is not limited to substrate release [11] and site-specific interactions (VIMP). Conceivably, the regulatory role of the D1 domain does not require heavy-duty ATP hydrolysis but demands precise control and timing. One way to accomplish this task is to include an additional nucleotide state such as the ADP-locked state, allowing more precise control of N domain conformation and imposing asymmetry. One consequence of failure to properly regulate the D1 domain function of p97 is MSP. MSP mutant p97 has a reduced ADP-binding affinity, a lower amount of prebound ADP and un-coordinated movement of the N domain [13, 44]. In the present study, we showed that full-length MSP mutants lose the ability to modulate their interaction with VIMP by ATP, further supporting the hypothesis of weakened control of the N domain conformation by the D1 domain in these mutants. As a result, pathogenic p97 mutants can constitutively interact with VIMP during the ATP hydrolysis cycle and conceivably fail to coordinate with the conformational changes generated by the D2 domains, leading to ERAD substrate accumulation and age-dependent tissue damage as a result of cellular stress [9, 10].

## Materials and Methods

### Plasmid construction, protein expression and purification of p97 and VIMP variants

Expression and purification of full-length (residues 1−806), N-D1 short (residues 1−460) and N-D1 long (residues 1−480) p97 fragments were done as described previously [13, 42, 44]. Construction of the GST-VIMPc fusion plasmid (pET42-GST-VIMPc) was previously reported [34]. Variants of GST-VIMP constructs were generated by introducing a stop codon at different positions using a QuikChange site-directed mutagenesis kit (Aglient Technology, Santa Cruz, CA, USA). VIMPx (pET42-His-VIMP) was constructed by introducing a stop codon after residue M122, and replacing the GST-tag with a hexahistidine-tag on the pET42-GST-VIMPc.

The pET42-His-VIMPx plasmid was transformed into *Escherichia  coli* BL21(DE3) for expression. Cells were grown at 37 ^o^C until OD_600_ reached ~1.0. A final 1 mM isopropyl B-D-1-thiogalactopyranoside (IPTG) was then added to induce VIMPx expression at 25 ^o^C for 20 h. Cells were harvested, resuspended in Buffer A (25 mM Tris, pH 7.5, 0.3 M NaCl, 10% glycerol) supplemented with 50 mM imidazole and a protease-inhibitor cocktail (Sigma-Aldrich, St Louis, MO, USA), and disrupted by sonication. Cell debris was removed by centrifugation at 15 000×*g* at 4 ^o^C for 30 min. The supernatant was incubated with Ni-NTA resin (Qiagen, Valencia, CA, USA) pre-equilibrated with Buffer A supplemented with 50 mM imidazole for 1 h at 4 ^o^C with mixing. The resin was washed with Buffer A supplemented with 100 mM imidazole and VIMPx was eluted with Buffer A supplemented with 250 mM imidazole. Fractions with VIMPx were pooled and concentrated using an Amicon Ultra-4 centrifugal filter unit (Millipore, Billerica, MA, USA). The concentrated sample was further purified by running it through a Superdex 75 size exclusion chromatographic column with a buffer containing 25 mM Tris, pH 8.0, 200 mM NaCl. The fractions were pooled, concentrated and dialyzed against 25 mM Tris, pH 8.0, 150 mM NaCl.

The selenomethionine derivative VIMPx (^SeMet^VIMPx) was obtained by growing cells in M9 minimal media. Amino acids including selenomethionine were added when OD_600_ reached ~1. A final 1 mM isopropyl B-D-1-thiogalactopyranoside (IPTG) was added to the culture media for induction of expression 15 min after the addition of amino acids, which were allowed to grow at 25 ^o^C for a further 20 h. Cells were harvested, resuspended in Buffer B (25 mM Tris, pH 7.5, 0.3 M NaCl, 2 mM EDTA, 2 mM DTT, 10% glycerol) supplemented with 50 mM imidazole and a protease-inhibitor cocktail (Sigma-Aldrich), and disrupted by sonication. Purification procedures similar to those for the native protein were used, except that 2 mM DTT was added during all purification steps and cOmplete His-Tag Purification Resin (Roche, Indianapolis, IN, USA) was used.

### Crystallization and X-ray diffraction data collection

All crystals were grown using the sitting-drop vapor diffusion method at 16 ^o^C. Crystallization of VIMPx was set up by mixing a protein solution of 14 mg/ml with a well solution containing 0.1 M 2-(N-morpholino)ethanesulfonic acid (MES), pH 6.0, 20% 2-propanol, 20% polythylene glycol monomethyl ether (PEG MME) 2000 in 1:1 ratio. Needle crystals were cryoprotected with the well solution, with glycerol concentration increased to 25% stepwise and flash-cooled in liquid propane.

For crystals of the p97-VIMPx complex, 25 μl ^N-D1^p97 protein solution of 7 mg/ml was mixed with MgCl_2_ and AMP-PNP to a final concentration of 40 and 4 mM, respectively, and incubated on ice for 30 min. Then, 4 μl of VIMPx (11 mg/ml) was added and incubated at room temperature with mixing for 30 min and then spun down at 18 000×*g* for 30 min at 16 ^o^C. The supernatant was used to set up crystallization. Crystals of ^ND1^p97^L198W^-VIMPx and ^ND1^p97^A232E^-^SeMet^VIMPx were grown by mixing the admixture prepared above with well solution containing 0.1 M Tris, pH 8, 15% ethanol, 100 mM NaCl, 7% 2-methyl-2,4-pentanediol (MPD) in a 1:1 ratio. Crystals of ^ND1^p97^A232E^-VIMPx and ^ND1^p97^A232E^-^SeMet^VIMPx were grown by mixing the admixture with well solution containing 0.1 M Tris, pH 8, 6–7% ethanol, 100 mM NaCl, 3.6–4.2% 2-methyl-2,4-pentanediol (MPD) in a 1:1 ratio. The crystals were cryoprotected with the well solution supplemented with 15% PEG400 and flash-cooled in liquid propane.

X-ray diffraction experiments were carried out at 100 K at the SER-CAT beamline of the Advanced Proton Source (APS) at Argonne National Laboratory, Argonne, IL, USA. Diffraction images were recorded with MarCCD detectors (Raynonix, Evanston, IL, USA), and processed and scaled with the HKL2000 package (HKL research, Charlottesville, VA, USA) [50].

### Structure determination

The crystal structures of VIMPx and the p97-VIMPx complex were determined by molecular replacement with the program Phaser [51] using PDB:2Q2F and PDB:4KO8 [44] as search models, respectively. The following crystal structures were determined: (1) the ^ND1^p97^A232E^-VIMPx complex was crystallized with the symmetry of the *P*622 space group, having one VIMPx molecule and one p97 subunit per asymmetric unit. (2) Crystals of ^ND1^p97^A232E^ in complex with ^SeMet^VIMPx, grown in the presence of AMP-PNP, have the same space group as crystals of the ^ND1^p97^A232E^-VIMPx complex. A data set was obtained to 3.75 Å resolution. (3) The crystals obtained for the p97 mutant L198W (^ND1^p97^L198W^) in complex with VIMPx diffracted X-rays to a lower resolution of 3.41 Å. This crystal also has the space group symmetry of *P*622 but has a larger unit cell. All structures were refined using Refmac [52] in the CCP4 program package [53]. All structural models were manually built using the program COOT [54].

### VIMP depletion, p97 membrane binding and pull-down assays

Dog pancreas microsomes [34] were solubilized in a buffer containing 1% deoxyBigCHAP followed by centrifugation to remove insoluble materials. The supernatant fractions were repeatedly incubated with Protein A beads containing anti-VIMP antibodies three times. The resulting unbound fractions were analyzed by immunoblotting (Figure 1a).

We mixed 2 μg p97 with 15 μl ER microsomes in a buffer 30 μl in volume and kept the reaction on ice for 20 min. The reaction mixture was then layered on top of a buffer (50 mM HEPES pH 7.3, 150 mM potassium acetate and 10 mM magnesium acetate) containing 40% sucrose. The samples were centrifuged at 100 000×*g* for 20 min and the membrane pellets were washed and then solubilized in sample buffer for analysis.

Variants of GST-VIMP were first incubated with glutathione resin (GenScript, Piscataway, NJ, USA) in a binding buffer (20 mM Tris, pH 8.0, 150 mM NaCl, 3 mM MgCl_2_, 10% glycerol, 0.1% Triton X-100) for 1 h at 4 ^o^C. The beads were recovered, washed three times with binding buffer and then incubated with p97 proteins (full-length or N-D1 (residues 1−480)) in the binding buffer supplemented with 2 mM nucleotide for 2 h at 4 ^o^C. The beads were then washed three times with binding buffer containing 2 mM nucleotide. Proteins that were retained on beads were released with sodium dodecyl sulfate–polyacrylamide gel electrophoresis sample buffer and analyzed by sodium dodecyl sulfate–polyacrylamide gel electrophoresis .

### ATPase activity assay

The ATPase activity of ^FL^p97 was determined as previously described [42]. Various concentrations of His-VIMPc were mixed with p97 before the substrate ATP was added to initiate the hydrolysis reaction.

### Determination of VIMPx binding affinity to p97 by isothermal titration calorimetry

Isothermal titration calorimetry experiments were performed using an iTC200 calorimeter (Malvern Instruments, Malvern, UK). All protein samples were dialyzed against assay buffer (50 mM Tris, pH 8.0, 150 mM NaCl, 2 mM MgCl_2_, 5% glycerol) overnight. Samples were passed through a 0.22 μm spin filter, and final protein concentration was then determined by optical absorption at A_280_ using the extinction coefficients 7450, 21430, 35870 and 5500 M^−1^cm^−1^ for ^N^p97, ^ND1^p97 (residue 1−480), ^FL^p97 and VIMPx, respectively. All titrations were performed in the assay buffer at 25 ^o^C. A final 2 mM of ADP or AMP-PNP was added to both p97 and VIMPx before the titration experiments. A heat exchange background because of dilution was determined by titrating VIMPx to buffer alone and subsequently subtracted from each experimental thermogram. Data were fit using a one-site model using Origin7 software (OriginLab, Northampton, MA, USA). The average binding parameters were obtained from three independent experiments.

### Accession codes

Atomic coordinates and structure factors have been deposited in the Protein Data Bank, under accession codes 5KIU (VIMPx), 5KIW (^ND1^p97^L198W^-VIMPx) and 5KIY (^ND1^p97^A232E^-VIMPx).

## Figures and Tables

**Table 1:** Statistics on the quality of diffraction data sets and refined atomic models

	***VIMPx***	^***ND1***^***p97***^***L198W***^***- VIMPx***	^***ND1***^***p97*** *^A^*^***232E***^***- VIMPx***	^***ND1***^***p97*** *^A^*^***232E***^***-***^***SeMet***^***VIMPx***
Bound nucleotide	None	AMP-PNP	AMP-PNP	AMP-PNP
				
*Data collection*				
Space group	*C*2	*P*622	*P*622	*P*622
Unit cell (*a, b, c*, Å) * *(*α, β, γ,* °)	121.7, 18.0, 34.0 90.0, 95.6, 90.0	153.8, 153.8, 240.6 90.0, 90.0, 120.0	145.0, 145.0, 119.8 90.0, 90.0, 120.0	143.9, 143.9, 119.3 90.0, 90.0, 120.0
Resolution (Å)	50–2.20 (2.30–2.20)^a^	50–3.40 (3.48–3.40)	50–2.79 (2.89–2.79)	50–3.80 (3.94–3.80)
R_merge_^b^ (%)	9.1 (33.9)	11.8 (36.2)	8.0 (77.9)	11.8 (33.0)
Completeness (%)	97.6 (92.5)	97.2 (79.7)	98.3 (85.6)	90.2 (78.2)
Total observation	11 211	197 220	159 420	62 379
Unique reflections	3 854	23 015	18 668	6 980
I/σ (I)	11.7 (2.5)	12.3 (1.7)	22.8 (1.0)	12.8 (2.4)
*R*_*pim*_	0.058 (0.214)	0.038 (0.234)	0.027 (0.403)	0.033 (0.148)
CC (1/2)	0.988 (0.705)	0.995 (0.157)	0.999 (0.227)	0.996 (0.556)
				
*Refinement statistics*				
Resolution (Å)	2.20	3.41	2.79	
R_free_ (%)	26.2 (28.9)	29.3 (49.7)	25.3 (46.9)	
R_work_ (%)	20.7 (25.6)	23.3 (47.7)	19.0 (42.5)	
Rmsd bond length (Å)	0.011	0.016	0.016	
Rmsd bond angle (°)	1.33	1.94	1.90	
Coordinate error (Å)	0.25	0.49	0.34	
NCS symmetry	1	2	1	
Number of non-H atoms	643	7 845	3 887	
Number of residues	76	991	487	
Number of solvent atoms	12	1	21	
Number of AMP-PNP	—	2	1	
Number of Mg^2+^ ions	—	2	1	
				
*Ramachandran analysis*				
Most favored (%)	100	88.9	90.0	
Allowed (%)	0	10.8	10.0	
Generously allowed (%)	0	0.3	0	
Disallowed (%)	0	0	0	
				
PDB code	5KIU	5KIW	5KIY	

a Values in parentheses are for the highest resolution shells.

b R_merge_ is defined as Σ|**I**_**h,i**_ - <**I**_**h**_>| / Σ**I**_**h,i**_, where **I**_**h,i**_ is the intensity for **i**^th^ observation of a reflection with Miller index **h**, and <**I**_**h**_> is the mean intensity for all measured **I**_**h**_s and Friedel pairs.

**Table 2:** Rotational (°) and translational (Å) movements measured for the N domain in AMP-PNP-bound and in ATPγS-bound forms in the N-D1 fragment of p97 in relation to the ADP-bound form^a^

***Bound nucleotide***	***VIMP binding***	***PDB code***	***Chain***		***Angle***	***Distance***
		5KIW	A		9.8	6.4
AMP-PNP	Yes	5KIW	B		19.4	11.3
		5KIY	A		15.9	9.6
				Mean	15.0±4.9	9.1±2.5
		1E32	A		0.0	0.0
ADP	No	5DYG	A		0.5	0.3
		4KOD	A		1.4	0.58
		5DYI	A		1.3	0.58
				Mean	0.8±0.7	0.4±0.3
		3HUI	A		22.6	12.8
ATPγS	No	3HU2	A		21.7	12.1
		4KO8	A		20.5	11.7
		4KLN	A		21.1	12.2
				Mean	21.5±0.9	12.2±0.5

a All structures were aligned with Cα atoms of p97 D1 domain (PDB:1E32, residues 210–370). Residue G207 was identified as the reference point (Point A fixed in all alignments in Figure 5c) for subsequent calculations. The center of mass (COM) of the N domain (residues 30–180) in each structure was calculated. The COM in 1E32 was set as Point B to represent the Down-conformation, and the COMs in other structures were set as Points C. The translational movement of the N domain was quantified by measuring the distance from Point B to Point C, and the angular movement (α) is defined as the angle between lines AB and AC.

## References

[bib1] Vembar SS, Brodsky JL. One step at a time: endoplasmic reticulum-associated degradation. Nat Rev Mol Cell Biol 2008; 9: 944–957. 19002207 10.1038/nrm2546 PMC2654601

[bib2] Meyer H, Weihl CC. The VCP/p97 system at a glance: connecting cellular function to disease pathogenesis. J Cell Sci 2014; 127: 3877–3883. 25146396 10.1242/jcs.093831 PMC4163641

[bib3] Christianson JC, Ye Y. Cleaning up in the endoplasmic reticulum: ubiquitin in charge. Nat Struct Mol Biol 2014; 21: 325–335. 24699081 10.1038/nsmb.2793 PMC9397582

[bib4] Ye Y, Shibata Y, Kikkert M, van Voorden S, Wiertz E, Rapoport TA. Recruitment of the p97 ATPase and ubiquitin ligases to the site of retrotranslocation at the endoplasmic reticulum membrane. Proc Natl Acad Sci USA 2005; 102: 14132–14138. 16186510 10.1073/pnas.0505006102 PMC1242302

[bib5] Lilley BN, Ploegh HL. Multiprotein complexes that link dislocation, ubiquitination, and extraction of misfolded proteins from the endoplasmic reticulum membrane. Proc Natl Acad Sci USA 2005; 102: 14296–14301. 16186509 10.1073/pnas.0505014102 PMC1242303

[bib6] Christianson JC, Olzmann JA, Shaler TA et al. Defining human ERAD networks through an integrative mapping strategy. Nat Cell Biol 2012; 14: 93–105. 10.1038/ncb2383 PMC3250479 22119785

[bib7] DeLaBarre B, Brunger AT. Complete structure of p97/valosin-containing protein reveals communication between nucleotide domains. Nat Struct Biol 2003; 10: 856–863. 12949490 10.1038/nsb972

[bib8] Ye Y, Meyer HH, Rapoport TA. Function of the p97-Ufd1-Npl4 complex in retrotranslocation from the ER to the cytosol: dual recognition of nonubiquitinated polypeptide segments and polyubiquitin chains. J Cell Biol 2003; 162: 71–84. 12847084 10.1083/jcb.200302169 PMC2172719

[bib9] Weihl CC, Dalal S, Pestronk A, Hanson PI. Inclusion body myopathy-associated mutations in p97/VCP impair endoplasmic reticulum-associated degradation. Hum Mol Genet 2006; 15: 189–199. 16321991 10.1093/hmg/ddi426

[bib10] Erzurumlu Y, Kose FA, Gozen O, Gozuacik D, Toth EA, Ballar P. A unique IBMPFD-related P97/VCP mutation with differential binding pattern and subcellular localization. Int J Biochem Cell Biol 2013; 45: 773–782. 23333620 10.1016/j.biocel.2013.01.006

[bib11] Bodnar NO, Rapoport TA. Molecular mechanism of substrate processing by the Cdc48 ATPase complex. Cell 2017; 169: 722–735. 28475898 10.1016/j.cell.2017.04.020 PMC5751438

[bib12] Blythe EE, Olson KC, Chau V, Deshaies RJ. Ubiquitin- and ATP-dependent unfoldase activity of P97/VCP*NPLOC4*UFD1L is enhanced by a mutation that causes multisystem proteinopathy. Proc Natl Acad Sci USA 2017; 114: E4380–E4388. 28512218 10.1073/pnas.1706205114 PMC5465906

[bib13] Tang WK, Li D, Li CC et al. A novel ATP-dependent conformation in p97 N-D1 fragment revealed by crystal structures of disease-related mutants. EMBO J 2010; 29: 2217–2229. 20512113 10.1038/emboj.2010.104 PMC2905243

[bib14] Zalk R, Shoshan-Barmatz V. ATP-binding sites in brain p97/VCP (valosin-containing protein), a multifunctional AAA ATPase. Biochem J 2003; 374: 473–480. 12747802 10.1042/BJ20030219 PMC1223595

[bib15] Latterich M, Frohlich KU, Schekman R. Membrane fusion and the cell cycle: Cdc48p participates in the fusion of ER membranes. Cell 1995; 82: 885–893. 7553849 10.1016/0092-8674(95)90268-6

[bib16] Acharya U, Jacobs R, Peters JM, Watson N, Farquhar MG, Malhotra V. The formation of Golgi stacks from vesiculated Golgi membranes requires two distinct fusion events. Cell 1995; 82: 895–904. 7553850 10.1016/0092-8674(95)90269-4

[bib17] Ramanathan HN, Ye Y. The p97 ATPase associates with EEA1 to regulate the size of early endosomes. Cell Res 2012; 22: 346–359. 21556036 10.1038/cr.2011.80 PMC3271578

[bib18] Richly H, Rape M, Braun S, Rumpf S, Hoege C, Jentsch S. A series of ubiquitin binding factors connects CDC48/p97 to substrate multiubiquitylation and proteasomal targeting. Cell 2005; 120: 73–84. 15652483 10.1016/j.cell.2004.11.013

[bib19] Ye Y, Meyer HH, Rapoport TA. The AAA ATPase Cdc48/p97 and its partners transport proteins from the ER into the cytosol. Nature 2001; 414: 652–656. 11740563 10.1038/414652a

[bib20] Braun S, Matuschewski K, Rape M, Thoms S, Jentsch S. Role of the ubiquitin-selective CDC48(UFD1/NPL4 )chaperone (segregase) in ERAD of OLE1 and other substrates. EMBO J 2002; 21: 615–621. 11847109 10.1093/emboj/21.4.615 PMC125867

[bib21] Song EJ, Yim SH, Kim E, Kim NS, Lee KJ. Human Fas-associated factor 1, interacting with ubiquitinated proteins and valosin-containing protein, is involved in the ubiquitin-proteasome pathway. Mol Cell Biol 2005; 25: 2511–2524. 15743842 10.1128/MCB.25.6.2511-2524.2005 PMC1061599

[bib22] Kondo H, Rabouille C, Newman R et al. p47 is a cofactor for p97-mediated membrane fusion. Nature 1997; 388: 75–78. 9214505 10.1038/40411

[bib23] Hanzelmann P, Schindelin H. Characterization of an additional binding surface on the p97 N-Terminal domain involved in bipartite cofactor interactions. Structure 2016; 24: 140–147. 26712280 10.1016/j.str.2015.10.027

[bib24] Hanzelmann P, Buchberger A, Schindelin H. Hierarchical binding of cofactors to the AAA ATPase p97. Structure 2011; 19: 833–843. 21645854 10.1016/j.str.2011.03.018

[bib25] Dreveny I, Kondo H, Uchiyama K, Shaw A, Zhang X, Freemont PS. Structural basis of the interaction between the AAA ATPase p97/VCP and its adaptor protein p47. EMBO J 2004; 23: 1030–1039. 14988733 10.1038/sj.emboj.7600139 PMC380986

[bib26] Madsen L, Seeger M, Semple CA, Hartmann-Petersen R. New ATPase regulators--p97 goes to the PUB. Int J Biochem Cell Biol 2009; 41: 2380–2388. 19497384 10.1016/j.biocel.2009.05.017

[bib27] Kim KH, Kang W, Suh SW, Yang JK. Crystal structure of FAF1 UBX domain in complex with p97/VCP N domain reveals a conformational change in the conserved FcisP touch-turn motif of UBX domain. Proteins 2011; 79: 2583–2587. 21739474 10.1002/prot.23073

[bib28] Lim JJ, Lee Y, Ly TT et al. Structural insights into the interaction of p97 N-terminus domain and VBM motif in rhomboid protease, RHBDL4. Biochem J 2016; 473: 2863–2880. 27407164 10.1042/BCJ20160237

[bib29] Ewens CA, Panico S, Kloppsteck P et al. The p97-FAF1 protein complex reveals a common mode of p97 adaptor binding. J Biol Chem 2014; 289: 12077–12084. 24619421 10.1074/jbc.M114.559591 PMC4002113

[bib30] Pye VE, Beuron F, Keetch CA et al. Structural insights into the p97-Ufd1-Npl4 complex. Proc Natl Acad Sci USA 2007; 104: 467–472. 17202270 10.1073/pnas.0603408104 PMC1761865

[bib31] Shchedrina VA, Zhang Y, Labunskyy VM, Hatfield DL, Gladyshev VN. Structure-function relations, physiological roles, and evolution of mammalian ER-resident selenoproteins. Antioxid Redox Signal 2010; 12: 839–849. 19747065 10.1089/ars.2009.2865 PMC2864662

[bib32] Christensen LC, Jensen NW, Vala A et al. The human selenoprotein VCP-interacting membrane protein (VIMP) is non-globular and harbors a reductase function in an intrinsically disordered region. J Biol Chem 2012; 287: 26388–26399. 22700979 10.1074/jbc.M112.346775 PMC3406722

[bib33] Liu J, Li F, Rozovsky S. The intrinsically disordered membrane protein selenoprotein S is a reductase *in vitro*. Biochemistry 2013; 52: 3051–3061. 23566202 10.1021/bi4001358 PMC3675161

[bib34] Ye Y, Shibata Y, Yun C, Ron D, Rapoport TA. A membrane protein complex mediates retro-translocation from the ER lumen into the cytosol. Nature 2004; 429: 841–847. 15215856 10.1038/nature02656

[bib35] Stapf C, Cartwright E, Bycroft M, Hofmann K, Buchberger A. The general definition of the p97/valosin-containing protein interacting motif (VIM) delineates a new family of p97 co-factors. J Biol Chem 2011; 286: 38670–38678. 21896481 10.1074/jbc.M111.274472 PMC3207395

[bib36] Hanzelmann P, Schindelin H. The structural and functional basis of the p97/valosin-containing protein (VCP)-interacting motif (VIM): mutually exclusive binding of cofactors to the N-terminal domain of p97. J Biol Chem 2011; 286: 38679–38690. 21914798 10.1074/jbc.M111.274506 PMC3207442

[bib37] Lee JH, Kwon JH, Jeon YH, Ko KY, Lee SR, Kim IY. Pro178 and Pro183 of selenoprotein S are essential residues for interaction with p97(VCP) during endoplasmic reticulum-associated degradation. J Biol Chem 2014; 289: 13758–13768. 24700463 10.1074/jbc.M113.534529 PMC4022850

[bib38] Schuller JM, Beck F, Lossl P, Heck AJ, Forster F. Nucleotide-dependent conformational changes of the AAA+ ATPase p97 revisited. FEBS Lett 2016; 590: 595–604. 26849035 10.1002/1873-3468.12091

[bib39] Banerjee S, Bartesaghi A, Merk A et al. 2.3A resolution cryo-EM structure of human p97 and mechanism of allosteric inhibition. Science 2016; 351: 871–875. 26822609 10.1126/science.aad7974 PMC6946184

[bib40] Schuetz AK, Kay LE. A dynamic molecular basis for malfunction in disease mutants of p97/VCP. Elife 2016; 5: e20143. 27828775 10.7554/eLife.20143 PMC5102582

[bib41] Davies JM, Tsuruta H, May AP, Weis WI. Conformational changes of p97 during nucleotide hydrolysis determined by small-angle X-Ray scattering. Structure 2005; 13: 183–195. 15698563 10.1016/j.str.2004.11.014

[bib42] Tang WK, Xia D. Role of the D1-D2 linker of human VCP/p97 in the asymmetry and ATPase activity of the D1-domain. Sci Rep 2016; 6: 20037. 26818443 10.1038/srep20037 PMC4730245

[bib43] Davies JM, Brunger AT, Weis WI. Improved structures of full-length p97, an AAA ATPase: implications for mechanisms of nucleotide-dependent conformational change. Structure 2008; 16: 715–726. 18462676 10.1016/j.str.2008.02.010

[bib44] Tang WK, Xia D. Altered intersubunit communication is the molecular basis for functional defects of pathogenic p97 mutants. J Biol Chem 2013; 288: 36624–36635. 24196964 10.1074/jbc.M113.488924 PMC3868774

[bib45] Chou TF, Bulfer SL, Weihl CC et al. Specific inhibition of p97/VCP ATPase and kinetic analysis demonstrate interaction between D1 and D2 ATPase domains. J Mol Biol 2014; 426: 2886–2899. 24878061 10.1016/j.jmb.2014.05.022 PMC4102644

[bib46] Xia D, Tang WK, Ye Y. Structure and function of the AAA+ ATPase p97/Cdc48p. Gene 2016; 583: 64–77. 26945625 10.1016/j.gene.2016.02.042 PMC4821690

[bib47] Zhang X, Gui L, Zhang X et al. Altered cofactor regulation with disease-associated p97/VCP mutations. Proc Natl Acad Sci USA 2015; 112: E1705–E1714. 25775548 10.1073/pnas.1418820112 PMC4394316

[bib48] Meyer HH, Kondo H, Warren G. The p47 co-factor regulates the ATPase activity of the membrane fusion protein, p97. FEBS Lett 1998; 437: 255–257. 9824302 10.1016/s0014-5793(98)01232-0

[bib49] Bulfer SL, Chou TF, Arkin MR. p97 disease mutations modulate nucleotide-induced conformation to alter protein-protein interactions. ACS Chem Biol 2016; 11: 2112–2116. 27267671 10.1021/acschembio.6b00350 PMC5224236

[bib50] Otwinowski Z, Minor W. Processing of X-ray diffraction data collected in oscillation mode. Methods Enzymol 1997; 276: 307–326. 10.1016/S0076-6879(97)76066-X 27754618

[bib51] Storoni LC, McCoy AJ, Read RJ. Likelihood-enhanced fast rotation functions. Acta Cryst Sec D Biol Cyst 2004; 60: 432–438. 10.1107/S0907444903028956 14993666

[bib52] Murshudov GN, Vagin AA, Dodson EJ. Refinement of macromolecular structures by the maximum-likelihood method. Acta Cryst Sec D, Biol Cryst 1997; 53: 240–255. 10.1107/S0907444996012255 15299926

[bib53] Collaborative Computational Project N. The CCP4 suite: programs for protein crystallography. Acta Cryst Sec D Biol Cryst 1994; 50: 760–763. 10.1107/S0907444994003112 15299374

[bib54] Emsley P, Cowtan K. Coot: model-building tools for molecular graphics. Acta Cryst Sec D Biol Cryst 2004; 60: 2126–2132. 10.1107/S0907444904019158 15572765

[bib55] Zhang X, Shaw A, Bates PA et al. Structure of the AAA ATPase p97. Mol Cell 2000; 6: 1473–1484. 11163219 10.1016/s1097-2765(00)00143-x

