# Supplementary material for: Structural basis for nucleotide-modulated p97 association with the ER membrane
Source: Cell Discov. 2017 Dec 12;3:17045–. doi: 10.1038/celldisc.2017.45 (PMC5725882; doi:10.1038/celldisc.2017.45)
Supplement: Supplementary Information [file celldisc201745-s1.pdf]

Supplemental Materials

Table S1. Rms deviations (Å) of pair-wise structural superposition between various ^{ND1}p97 structures[a]

		AMP-PNP			ATPγS				ADP			
		**5KIW Chain A**	**5KIW Chain B**	**5KIY Chain A**	**3HU1 Chain A**	**3HU2 Chain A**	**4KO8 Chain A**	**4KLN Chain A**	**1E32 Chain A**	**5DYG Chain A**	**4KOD Chain A**	**5DYI Chain A**
**AMP-PNP**	**5KIW Chain A**	---	2.815	2.230	3.353	3.219	3.057	3.077	8.831	8.772	8.839	8.879
	**5KIW Chain B**		---	1.134	1.068	0.973	0.850	0.815	9.449	9.404	9.431	9.495
	**5KIY Chain A**			---	1.481	1.378	1.242	1.250	9.305	9.253	9.292	9.353
**ATPγS**	**3HU1 Chain A**				---	0.416	0.928	0.760	9.010	9.855	9.894	9.948
	**3HU2 Chain A**					---	0.782	0.657	9.705	9.653	9.689	9.743
	**4KO8 Chain A**						---	0.689	9.458	9.403	9.437	9.499
	**4KLN Chain A**							---	9.638	9.583	9.616	9.672
**ADP**	**1E32 Chain A**								---	1.359	1.000	1.102
	**5DYG Chain A**									---	1.127	1.002
	**4KOD Chain A**										---	0.719
	**5DYI Chain A**											---

[a] rms deviations (Å) were determined by superposing Cα atoms of residues 21-460 of different structures. For structures that contain more than one subunit per asymmetric unit, only the indicated chain was used for calculation.

Table S2. Binding affinities of VIMPx to p97 variants determined by ITC[a.]

p97 variants	Nucleotide added	K_d (μM)	Stoichiometry (N)	ΔH ($kcal\ mol^{-1}$)	ΔS ($kcal\ mol^{-1}\ K^{-1}$)
^{N}p97	---	6.58 ± 0.41	1.27 ± 0.06	-3.0 ± 0.2	13.6 ± 0.7
^{ND1}p97^{wt}	ADP	6.21 ± 0.85	1.13 ± 0.06	-2.5 ± 0.2	15.3 ± 0.8
	AMP-PNP	5.52 ± 0.26	0.98 ± 0.05	-2.7 ± 0.1	15.0 ± 0.3
^{FL}p97^{wt}	ADP	ND[b]	ND[b]		
	AMP-PNP	7.00 ± 0.13	0.66 ± 0.08	-1.8 ± 0.1	17.5 ± 0.5

[a] All values are the average of at least three independent titrations.

[b] ND=Not determined. We did several runs for the same reaction but were unable to obtain a reliable value. This could be due to the combination of low binding stoichiometry in the presence of ADP, resulting in small amount of heat change generated by the interaction, and the low binding affinity of the interaction.

Table S3. Data sets and symmetries of crystals of the complex between VIMP and the long version of the p97 N-D1 fragment (1-480).

Data Set Name	Nucleotide State	Diffraction Resolution (Å)	NCS	Space Group
L198W(480)-PNP-VIMPx	AMP-PNP	3.4	2	*R*3
L198W(480)-γS-VIMPx	ATPγS	2.8	2	*P*6 (twined)

Supplemental Figure legends

Figure S1. Various experimental difference electron density maps (A) Difference Fourier map for bound AMP-PNP. The map, calculated with Fourier coefficients m*Fo*-*Fc* derived from refined structure of ^{ND1}p97^{A232E}-VIMPx, is represented in light-green mesh contoured at 3σ level and is fit with a stick model of AMP-PNP with carbon atoms colored yellow, oxygen red, nitrogen blue and phosphorous orange. The ribbon representation in blue is the D1 domain of the ^{ND1}p97^{A232E} and that in cyan is a neighboring D1 domain. (B) Difference Fourier map revealing bound VIMPx. The difference density derived from the ^{ND1}p97^{L198W}-VIMPx complex is shown as a light-green mesh contoured at 2.5σ level and is fit with a Cα tracing in cyan of a fragment of VIMPx. The N domain is represented by the ribbon diagram in magenta and its two subdomains are labeled. (C) Anomalous difference Fourier map, calculated at 4.0 Å resolution and contoured at 4σ level, is shown in gold mesh, which was assigned to the selenium atom of SeMet89 in VIMPx. The magenta ribbon represents the N domain of the ^{ND1}p97^{A232E} structure and the cyan Cα trace represents the VIMPx. (D) Stereo 2*Fo*-*Fc* map for VIMPx (chain C) of the ^{ND1}p97^{L198W}-VIMPx complex. The map is shown as a light-green mesh contoured at 1σ level fit with the VIMPx in cyan ribbon. The side-chains of the VIMPx residues are represented as stick models and labeled. (E) Cartoon representation of two hexameric ND1 rings after symmetry expansion of chains A and B, respectively, of the ^{ND1}p97^{L198W}-VIMPx structure. The N domains are colored in magenta and the D1 domains in blue. (F) The H1 helix of VIMPx in the ^{ND1}p97^{L198W}-VIMPx structure is stabilized by crystal contact with the N domain of a neighboring molecule. The N domain is shown as a molecular surface and the H1 helix is shown as a cartoon diagram.

Figure S2. Relative ATPase activity of ^{FL}p97, wild type and mutant, in the presence or absence of His-tagged VIMPc.

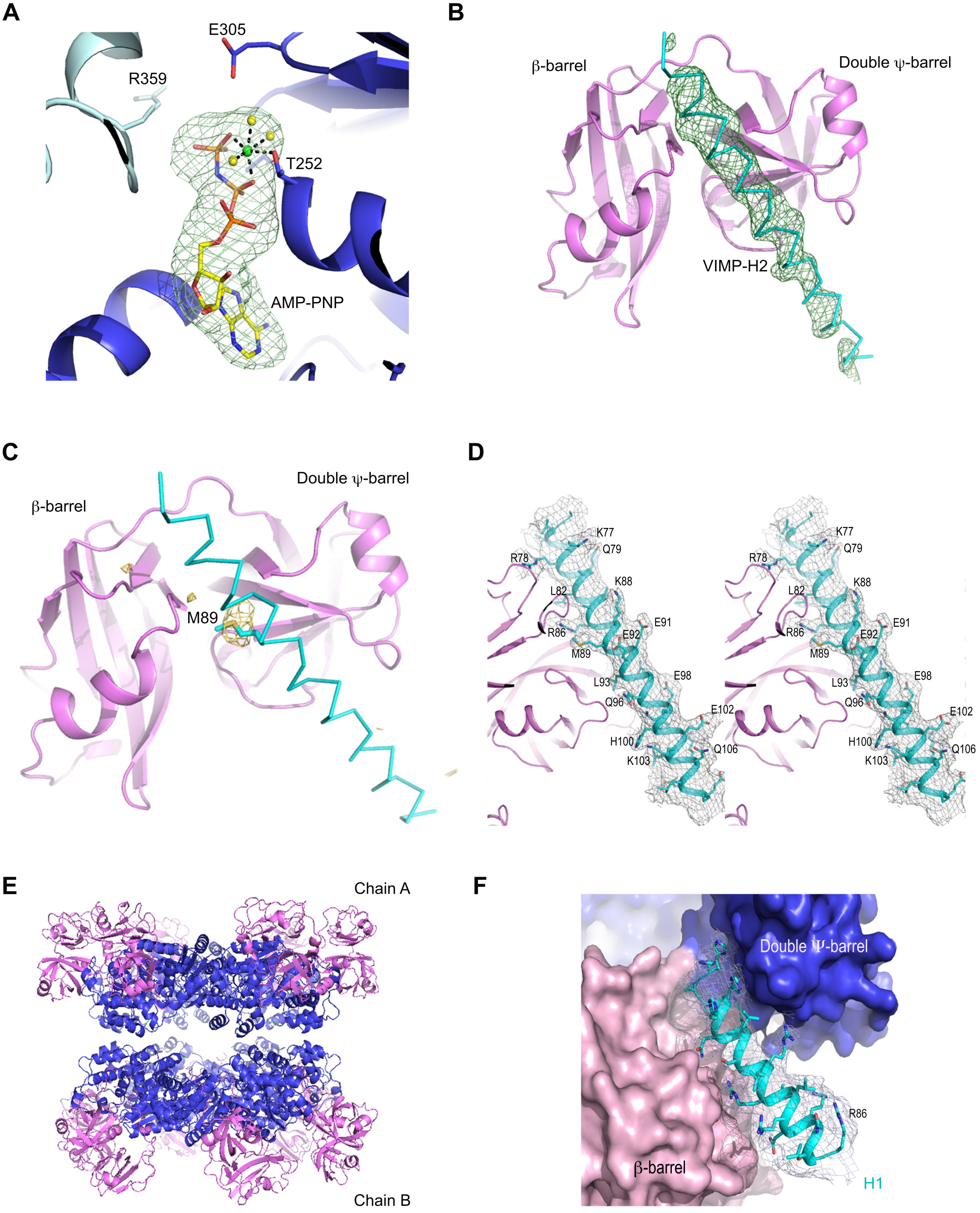

Tang et al., Figure S1

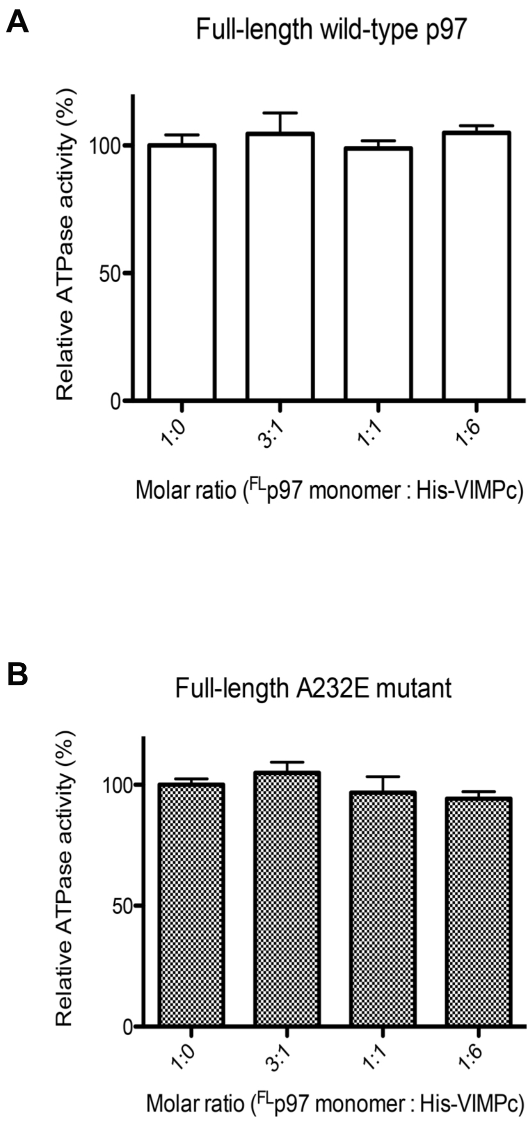

Tang et al. Figure S2
